# Advances of Patient-Derived Organoids in Personalized Radiotherapy

**DOI:** 10.3389/fonc.2022.888416

**Published:** 2022-04-29

**Authors:** Yuenan Wang, Ye Li, Zonghai Sheng, Weiwei Deng, Hongyan Yuan, Shubin Wang, Yajie Liu

**Affiliations:** ^1^ Department of Radiation Oncology, Peking University Shenzhen Hospital, Shenzhen, China; ^2^ Paul C. Lauterbur Research Center for Biomedical Imaging, Institute of Biomedical and Health Engineering, Shenzhen Institutes of Advanced Technology, Chinese Academy of Sciences, Shenzhen, China; ^3^ Department of Mechanical and Aerospace Engineering, Southern University of Science and Technology, Shenzhen, China; ^4^ Department of Medical Oncology, Peking University Shenzhen Hospital, Shenzhen, China

**Keywords:** patient-derived organoid (PDO), patient-derived xenograft (PDX), radiosensitizer, personalized medicine, radiotherapy

## Abstract

Patient-derived organoids (PDO), based on the advanced three-dimensional (3D) culture technology, can provide more relevant physiological and pathological cancer models, which is especially beneficial for developing and optimizing cancer therapeutic strategies. Radiotherapy (RT) is a cornerstone of curative and palliative cancer treatment, which can be performed alone or integrated with surgery, chemotherapy, immunotherapy, or targeted therapy in clinical care. Among all cancer therapies, RT has great local control, safety and effectiveness, and is also cost-effective per life-year gained for patients. It has been reported that combing RT with chemotherapy or immunotherapy or radiosensitizer drugs may enhance treatment efficacy at faster rates and lower cost. However, very few FDA-approved combinations of RT with drugs or radiosensitizers exist due to the lack of accurate and relevant preclinical models. Meanwhile, radiation dose escalation may increase treatment efficacy and induce more toxicity of normal tissue as well, which has been studied by conducting various clinical trials, very expensive and time-consuming, often burdensome on patients and sometimes with controversial results. The surged PDO technology may help with the preclinical test of RT combination and radiation dose escalation to promote precision radiation oncology, where PDO can recapitulate individual patient’ tumor heterogeneity, retain characteristics of the original tumor, and predict treatment response. This review aims to introduce recent advances in the PDO technology and personalized radiotherapy, highlight the strengths and weaknesses of the PDO cancer models, and finally examine the existing RT-related PDO trials or applications to harness personalized and precision radiotherapy.

## Introduction of PDO

Cancer is increasingly a major health problem worldwide. Developing novel cancer therapies offers opportunities to reduce the global cancer burden. Yet costs are quite high and success rates are quite low in clinical trials (1). The poor performance for new therapeutics or drug combinations to reach the clinic implies that many preclinical cancer models do not retain characteristics of the original tumor (2). Many anti-cancer therapeutics performing excellently *in vitro* and *in vivo* at the lab have failed in clinical trials (3). There is an emerging need for more relevant preclinical cancer models to faithfully reflect the original tumor behaviors of the individual patient.

The recently surged PDO technology, the 3D cell cultures derived from a patient’s tumor, may retain characteristics of the original tumor as a better *in vitro* cancer model. The PDO platform is thought to be the breakthrough moments with enormous potentials for cancer biology and personalized therapy as a preclinical human tumor model (4). Because the drug response in PDO models is positively correlated to that in patients, PDO becomes a great candidate to guide precision medicine (5). In fact, not only the response of tumor organoids *in vitro* to various anti-cancer agents is positively correlated with the *in vivo* response in mice and humans [observed by Vlachogiannis et al. (6) and Broutier et al. (7)], but also the genetic diversity, phenotypes and tumor heterogeneity are faithfully captured in PDO [reported by Weeber et al. (8) and Sachs et al. (9, 10)]. PDO models were reported to maintain the same chemoresistance, genetic mutations, and hypoxic gradients as those in the original tumor tissues by Miserocchi et al. (11), Jabs et al. (12), and Hubert et al. (13), respectively. Previously, Vlachogiannis et al. also found the PDO’s phenotypic and genotypic profiling possessed a high degree of similarity to the original patient’s tumor (6). It has been reported that PDO can also predict responses in metastatic colorectal cancer (CRC) patients, where the *in vitro* PDO model was developed from metastatic lesions to identify nonresponders to standard-of-care chemotherapy in a clinical study (14). In that prospective study, Ooft et al. showed the feasibility of generating PDO for evaluating chemotherapy sensitivity and predicted response of biopsied lesion in more than 80% of patients without misclassifying patients who would have benefited from treatment, which suggests that PDO model could be used to prevent cancer patients from undergoing ineffective irinotecan-based chemotherapy (14). Previous studies demonstrate that the PDO cancer model could predict patients’ response towards chemotherapy or radiotherapy (15, 16), complementing the existing treatment regimens to improve outcome and contribute to personalized cancer therapy (17).

In short, the PDO platform is a robust preclinical cancer model allowing tumor cells from individual patients to form the living, *in vitro*, 3D structured tissue culture. It can resemble features of the original tumor microenvironment while maintaining self-organize and self-renewal, described by Clevers and Drost (18). The PDO model not only facilitates discovering and developing novel therapeutics in cancer research (19), but also plays an important role in clinical decision-making for patients (20). Since they offer biological links with patient data, the PDO cancer models have emerged to an avatar for precision cancer therapy (21).

## Combined Radiation Strategies and Radiosensitizers

Radiotherapy (RT) has been a mainstay treatment for patients with cancer for a century (22). More than half of cancer patients undergo RT, either alone or together with surgery, chemotherapy, immunotherapy, targeted therapy, or hormone therapy. RT is used as an adjuvant therapy to combine with other therapeutic agents to achieve better outcomes with relatively low cost (23). Compelling evidences show that RT not only enables effective treatment of large numbers of cancer cases to save lives, but also brings positive economic benefits (24). Recent advances in intensity-modulated radiotherapy (IMRT) (25) and image guided radiotherapy (IGRT) (26) have substantially improved the accuracy and precision of RT delivery with better treatment outcomes, which makes RT an attractive and beneficial treatment modality for cancer patients (23). To further enhance treatment efficacy and increase tumor control, clinicians and researchers have explored the opportunities of RT combined with other therapeutics or radiosensitizers (27, 28).

For example, similar to radiosensitizing chemotherapy, the combination of RT and immunotherapy (IM) has great potential for radiosensitizing immunotherapy and synergistic effects to enhance the overall treatment efficacy (29). In fact, RT combined with IM has been considered as a paradigm shift for cancer treatment, and it is assumed that RT is most effective when it causes tumor-targeting immune response (28). The combined RT-IM strategy may have a systemic effect, improving the overall survival in patients with non-small-cell lung cancer (PACIFIC trial) (30). It is well known that radiation can evoke stimulatory or suppressive immune effects, yet the biological mechanisms underlying these effects are not completely understood. Radiation-associated immune stimulatory effects are complicated and they are highly individualized, depending on the tumor model, and radiation dose and fractionation. Therefore, the optimal combined RT-IM strategies may highly depend on patient selection, radiation dose distribution, treatment induced toxicity, individual patient’s radiosensitivity and immune response. In fact, it is crucial and difficult to select the right patient population and avoid unnecessary toxicity and cost in clinical decision-making (29, 30). The *in vitro* PDO models can help to not only investigate fundamental research of mechanisms underlying the RT-IM combination but also select right patients by conducting RT-drug screening on the relevant platform of personalized medicine.

In addition to radiosensitization induced by immunotherapy or synergistic targeted or cisplatin-based chemoradiation (31), metal-based nanoparticles (NP) such as gold NP have great promise to conquer intrinsic radioresistance and enhance RT efficacy in clinical applications (32). Unlike chemoresistance, radiation resistance may be a multifactorial phenomenon and has not been fully understood. Factors responsible for radioresistance include hypoxia or immune status, cancer stem cell persistence, p53 loss, or enhanced DNA repair, which highly depends on individual patient’s tumor characteristics and microenvironment (33). The classical lab models of immortal cell lines and *in vivo* animal models have been fundamental to radiobiological studies to date. However, there are limitations in those models of designing the effective combined RT-drug strategies or developing novel radiosensitizers (34). Considering the primary goal of RT has been increasing tumor control while reducing the probability of normal tissue side effects, a strategic translational program using new complementary preclinical models such as PDO is required to fulfill the knowledge gap in radiobiology research (34).

**Figure 1:**
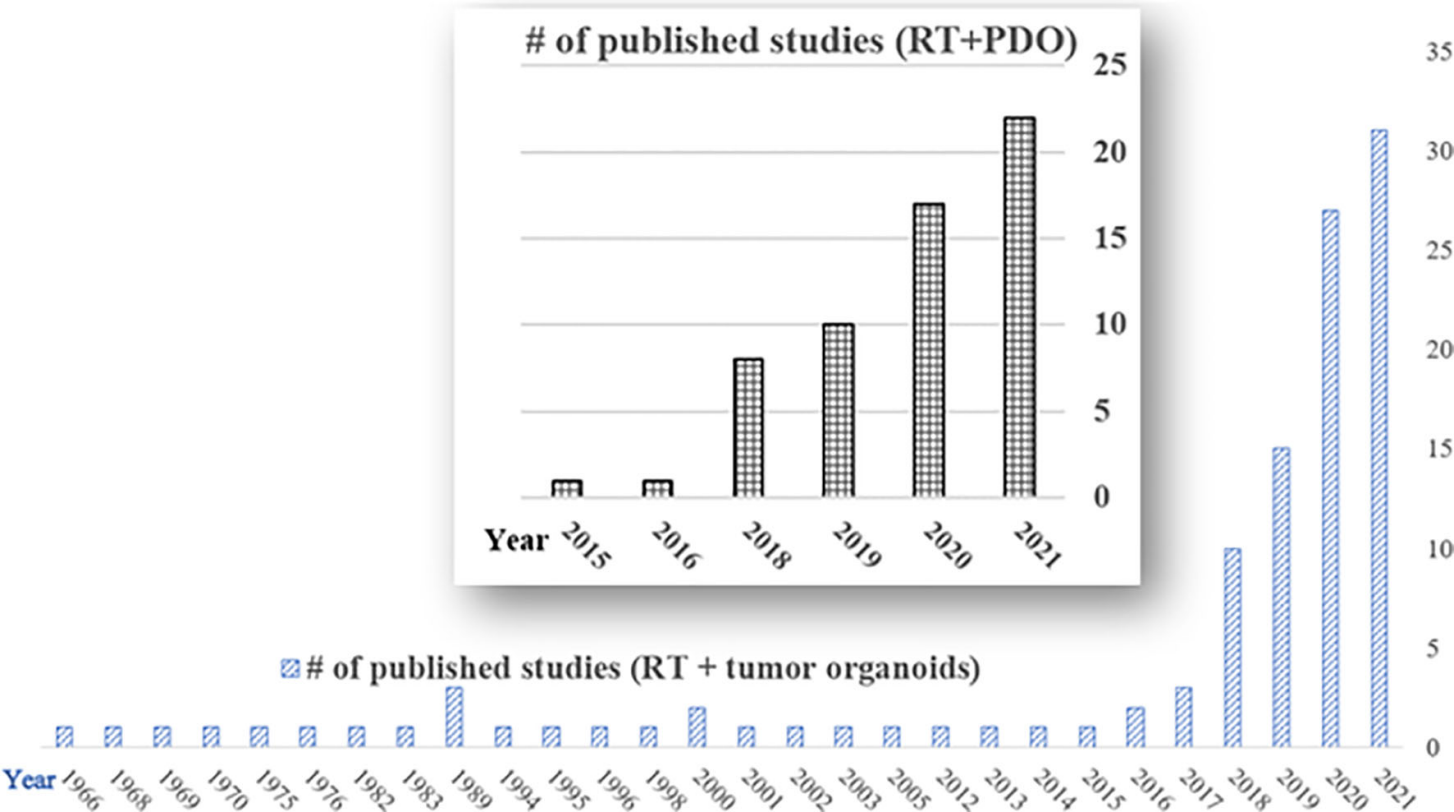
Publications of radiotherapy and tumor organoids on PubMed (till Dec 31, 2020). Total number of published studies of radiotherapy and tumor organoids (RT + tumor organoids) = 113; total number of published studies of radiotherapy and patient-derived organoids (RT+PDO) = 59. Notice there is a surge in the RT and PDO research area in the last four years (2018-2021).

With the development of chemotherapy, targeted therapy, immunotherapy, and radiosensitizers, the combined radiation strategies including RT regimens and sequences in the clinical setting become more and more complicated. RT combined with therapeutics or novel radiosensitizers needs a robust and personalized preclinical model, where organoids can be adopted to facilitate to predict individual’s response or the sensitivity to optimize radiation or drug dose for better clinical outcomes (35). From our preliminary literature search on PubMed till Dec 31, 2021, there are 113 studies in the field of RT and tumor organoids. There are 59 studies specifically about the PDO and RT, among which more than 90% were published during the last four years (**Figure 1**), demonstrating there’s a surge of research on applying PDO to the RT field, showing great promise to harness personalized and precision radiotherapy using the PDO platform.

## Comparisons of Preclinical Cancer Models

There are several commonly used preclinical cancer models, such as 2D *in vitro* adherent monolayer cell culture, 3D *in vitro* organoids including PDO, and *in vivo* animal models including PDX.

The traditional 2D adherent cancer cell lines, originally derived from primary patient materials, have contributed tremendously to the radiation biology research, including the clonogenic survival assay or screening for effective radiosensitizers (36). However, the 2D cell culture model cannot demonstrate cellular heterogeneity, cell-matrix and cell-cell interactions, which makes results in cell lines often overstate treatment responses and therefore the translation to clinical patient setting using 2D cell cultures becomes in convenient and inaccurate. The 2D cell cultures cannot represent genetic heterogeneity and the microenvironment of the original tumors. In addition, the cancer cell lines from primary patients may undergo extensive adaptation, therefore the 2D cell culture models can no longer represent treatment responses in real patients. Finally, it is impossible to study the long-term growth in 2D cell cultures.

A novel *in vitro* 3D culture technology leads to the development of organoids, as Sato et al. demonstrated that 3D epithelial organoids derived from small and large intestines could be established as long-term culture conditions and recapitulate structural and functional characteristics of the tissue of origin (37). Among tumor organoids, PDOs are derived from individual patient and can be expanded long term, cryopreserved, genetically and phenotypically stable, therefore they are suitable for many applications of cancer research (18). PDO can be developed collectively as different cancer biobanks for drug development, personalized medicine, and response evaluation (38). The PDO cancer model provides a unique opportunity for drug sensitivity testing, correlating with the genetic make-up of individual tumors, and modifying various dosing schemes *in vitro* to optimize treatment outcome of patients. Apparently, PDOs have advantages over traditional 2D cell cultures, making them suitable for biobanking and high-throughput drug or combined RT dose screening. As compared to animal models, organoids can reduce experimental complexity, and more importantly, enable the study of cancer and disease that are not easily or accurately modeled in animals (39). However, compared to 2D cell cultures, PDOs may grow with unpredictable and slow kinetics and the stiffness of extracellular matrix such as the Matrigel concentration can affect the organoid formation, differentiation, and drug response (39). Compared to animal models, PDO models lack stroma, blood vessels and immune cells. Nevertheless, the PDO model can represent individual patient’s genetic and phenotypic characteristics truthfully, has higher success rates of establishment and is less expensive than animal models.

The *in vivo* animal model can overcome many of the limitations of 2D cell cultures and 3D PDO models, but it is expensive, very time consuming, and cannot be completed during the RT treatment course of cancer patients. The animal model may be limited by the growing pressure of restricted use of animals in cancer research too. In preclinical cancer research, *in vitro* and *silico* findings should be confirmed *in vivo* prior to human translation, and hence animal models will remain crucial to the radiation oncology research. Also the commonly used subcutaneous tumor models in nude mice may not represent the real world’s tumor in patients. The orthotopic tumor model is assumed to be more accurate representing tumor-environmental interactions *in vivo*. However, the preservation of complexity also dramatically limits experimental control (40). Among the animal models, patient-derived tumor xenografts (PDX) are generated by transplanting freshly derived patient material subcutaneously or orthotopically into immunodeficient mice. The *in vivo* PDX model has the advantage of mimicking the biological characteristics and pathogenesis process of the original tumor more accurately than *in vitro* 2D cell culture, or 3D PDO culture, or *in vivo* animal models from cell lines. However, the PDX model has the limitations of limited engraftment efficiencies, time-consuming, and may undergo mouse-specific tumor evolution (40). Both PDO and PDX models maintain key features from their parental tumors of individual patients, which allows them to be used for a wide spectrum of cancer research. In contrast to PDX, PDO can be established and expanded with high efficiency from primary patient material. Compared to PDO culture, PDX can retain tumor-stroma interactions.

To summarize, the PDO model is less costly and less time-consuming than PDX or traditional animal models from cell lines, with reduced experimental complexity and feasible human disease modeling that is not easily or accurately realized in animal models (38). The clinical relevance and versatile feature of PDOs make this *in vitro* 3D organoid technology exciting to establish on a single patient basis for personalized oncology. On the other hand, there are intrinsic limitations of PDO, i.e., lack of stroma, blood vessels and immune cells, which may be addressed by developing co-culture systems in the future. Clevers et al. mentioned that the organoid approach may be not able to adapt from non-epithelial tumors (18). Nevertheless, the *in vitro* PDO model has emerged in personalized medicine to investigate cancer mechanisms, develop new drugs or combined therapies, predict normal tissue toxicity and radiosensitivity, conduct drug screening within a meaningful time window for patients (5, 14, 21, 41, 42).

## Personalized Radiotherapy With the PDO Models

### Published Studies of the PDO Models

Clinical studies using PDO cancer or normal tissue models with RT or the combined RT regimens are summarized in **Table 1**. Most of PDO models are preclinical cancer models, including lung cancer, esophageal cancer, colorectal cancer, locally advanced rectal cancer, pancreatic cancer, breast cancer, head and neck cancer, and glioblastoma. The last three lines of PDO models are based on patient-derived normal tissue organoids to investigate RT-induced injury or toxicity in the intestinal tract. The results from preclinical PDO cancer models show that PDOs can faithfully recapitulate genetical and functional features of the original tumor from individual patient, and hence radiosensitivity or the combined RT-drug sensitivity can be predicted for various cancer types. Meanwhile, if normal tissue from patients can be successfully modeled into PDOs, then RT-induced toxicity or injury can also be truthfully captured in the normal tissue PDO model. The cancer and normal tissue PDO models provide insights on personalized treatment regimens with evaluations of tumor control and radiation-induced toxicity for individual patient, which definitely helps to design the optimal combined RT strategies to increase overall survival and quality of life for cancer patients eventually.

**Table 1:** Summary of PDO cancer models or normal tissue models undergoing RT.

PDO types	Treatment regimen	Key findings	Sponsor	Reference
**Lung, colorectal and pancreatic adenocarcinoma**	RT=0,2,5Gy, 5-FU	PDO represented phenotypic and molecular heterogeneity in cancers. Therapeutic thresholds established using PDO growth rate and optical metabolic imaging to determine response to chemoRT. This study predicted individual patient’s sensitivity to chemo+RT.	University of Wisconsin, USA	Pasch 2019 (15)
**Locally advanced rectal cancer**	RT=8Gy, 5-FU, Irinotecan	PDO predicted treatment outcome of chemoRT	Fudan University, China	Yao 2020 (16)
**Rectal cancer**	RT=0-8Gy, 5-FU, FOLFOX	PDO showed clinically relevant chemo and RT responses	Memorial Sloan Kettering Cancer Center, USA	Ganesh 2019 (43)
**Rectal cancer**	RT=0,2,5Gy 5-FU, cetuximab	Cetuximab could potentiate RT based on KRAS mutational status and further mutations might impact cetuximab sensitivity. PDO could identify 5-FU/RT-resistant and assist proper personalized therapy	Medical University of South Carolina, USA	Janakiraman 2020 (44)
**Esophageal cancer**	γ/proton RT=5Gy, cisplatin, paclitaxel, FOLFOX, trametinib	PDO mirrors clinical response following neoadjuvant treatment and showed therapeutic values for individual patients with esophageal adenocarcinoma	University of Pennsylvania, USA	Karakasheva 2021 (45)
**Glioblastoma (GBM)**	RT=3Gy	PDO simultaneously cultured phenotypically diverse stem/non-stem GBM cells for stem cell biology/microenvironment	Cleveland Clinic, USA	Hubert 2016 (13)
**Pediatric and adult GBM**	RT=3Gy	PDO of GBM may offer a key approach to understand dynamic resistance mechanisms of cancer	Cleveland Clinic, USA	Sundar 2022 (46)
**GBM**	RT=10Gy, TMZ	PDO models presented a novel workflow for drug combination screening for more effective treatment for recurrent GBM	University of South Australia	Lenin 2021 (47)
**GBM**	RT=0, 5, 10 Gy	Cerebral organoids form rapidly and interconnect with tumor micro-tubes to invade normal host tissue, which provide a modeling system for primary GBM ex vivo for high-throughput drug screening	Cornell University, USA	Linkous 2019 (48)
**Head and neck squamous cell carcinoma**	RT=0-10Gy	HNSCC’s PDO recapitulated genetical, histological, and functional features for current and future therapy screening	University Medical Center Utrecht, Netherlands	Driehuis 2019 (17)
**HNSCC + CRC**	RT=1-10Gy	Medium-throughput drug screening using HNSCC and CRC adenocarcinoma organoids. Drug sensitivity parameters based on PDO models provided insight of being sensitive or resistant to a particular RT or chemo treatment	Crown Bioscience, Netherlands	Putker 2021 (49)
**Nasopharyngeal carcinoma (NPC)**	RT:0.2-30Gy cisplatin, 5-FU	Hypoxic NPC organoids were highly radioresistant and required a large RT dose to compensate for oxygen deficiency	A*STAR, Singapore	Lucky 2021 (35)
**Breast cancer**	RT~20Gy	PDO models were used to mirror radiation-induced cell recruitment. The complex *in vitro* PDO model is useful for tumor-stromal interactions, infiltration of immune cells and macrophage polarization within the irradiated microenvironment	Vanderbilt University, USA	Hacker 2020 (50)
**Small/large intestine organoids (normal tissue)**	RT=0-16Gy	Organoids show inherent radiosensitivity of small and large intestinal stem cells	Memorial Sloan Kettering Cancer Center, USA	Martin 2019 (42)
**Epithelial stem cell colonic/intestinal organoids**	RT=0-15, 30Gy	Different radioresistance in colonic epithelial stem cells vs. small intestinal stem cell	Fudan University, China	Hua 2017 (41)
**Rectal epithelial stem cell organoids**	RT=20Gy	Radiation-induced toxicity in rectal epithelial stem cell contributes to acute radiation injury in the rectum	University of Kansas Medical Center, USA	Tirado 2021 (51)

*RT, radiotherapy; 5-FU, 5-Fluorouracil; FOLFOX, 5-FU, leucovorin and oxaliplatin; temozolomide, TMZ.

### Ongoing Clinical Trials With the PDO Models

In addition to published studies described previously (**Table 1**), there are several ongoing clinical trials incorporating the organoids approach to evaluate the performance of therapeutics registered at the ClinictrialTrials.gov website. For example, in the FORESEE trial (functional precision oncology for metastatic breast cancer) sponsored by University of Utah, the PDO models are applied to monitor cancer recurrence risk upon individual undergoing personalized therapy (ClinicalTrials – NCT04450706). The PIONEER trial with the initiative of Precision Insights On N-of-1 Ex Vivo Effectiveness Research Based on Individual Tumor Ownership (Precision Oncology), sponsored by SpeciCare, planned to acquire 1000 samples on the PDO platform (Trial Identifier: NCT03896958). In the SYNCOPE trial, the sponsor of Helsinki University Central Hospital plans to conduct the Systemic Neoadjuvant and Adjuvant Control by Precision Medicine in Rectal Cancer (SYNCOPE) by conducting analysis of 93 organoids (NCT04842006). In the TUMOVASC trial sponsored by University Hospital, Strasbourg, France, 100 organoids samples were proposed to collect for high throughput screening device based on 3D nano-matrices and 3D tumors with functional vascularization (NCT04826913).

In addition to the four trials above, there are trials without acronyms registered at ClinicalTrials.gov for the organoid platform, such as “Novel 3D Myeloma Organoid to Study Disease Biology and Chemosensitivity” (Wake Forest University, USA, NCT03890614), “Feasibility Study of Multi-Platform Profiling of Resected Biliary Tract Cancer” (University of Washington, USA, NCT04561453), “The Culture of Ovarian Cancer Organoids and Drug Screening” (Chongqing University, China, NCT04768270), and “3D Bioprinted Models for Predicting Chemotherapy Response in Colorectal Cancer With/Without Liver Metastases” (Peking Union Medical College Hospital, China, NCT04755907).

Among the 32 studies in the “recruiting, enrolling, or active” status for the registered clinical trials found with the keywords “patient derived organoids” and “cancer, tumor, neoplasm” by March 15 2022 on the ClinicalTrial.gov website, there are 7 from United States covering lung, breast, pancreas, rectum cancers and cholangiocarcinoma; 6 from China covering gastric, colorectal, ovarian cancers and liver metastasis; 5 from Netherland covering glioma, pancreas, ovarian cancers and sarcoma; 3 from Singapore covering head and neck, gastrointestinal, breast, ovarian, neuroendocrine cancers; 2 from Germany covering gastric, esophageal, pancreas cancers; 2 from Canada covering breast, ovarian, pancreas, colorectal cancers; 1 from France for lung cancer; 1 from Switzerland for lung cancer; 1 from Denmark for pancreas cancer; 1 from Italy for ovarian cancer; 1 from Finland for colorectal cancer; 1 from Belgium for breast cancer; 1 from Hong Kong from meningioma. So far PDO trial data are limited to assess how well PDO performs in clinical settings. Still it is encouraging and exciting to see the increasing number of active clinical trials involving the organoids platform.

### Challenges of the PDO Models

The PDO models are not yet in the mainstream of cancer diagnosis and therapy. There are challenges facing the routine use of PDO in precision radiation oncology. The PDO method by definition requires viable tumor tissue, either from surgical resections or biopsies. The current practice of tissue handling requires rapid fixation for static histology or genomics tests. The tissue collecting of PDOs using fresh and viable tissue is relatively difficult compared to the standard tissue handling. In addition, it is also difficult for tumor tissue samples to be stored in a viable situation. Notice that tissue physiological and pathological signalings can change if kept in different environments. Therefore, optimizing and standardizing storage and handling conditions can be extremely important for the success rates of PDO establishment.

Another challenge is obtaining sufficient tissue cells from individuals. For solid tumors, large resections are relatively rare in the setting of metastatic disease, in core needle biopsies, fine-needle aspirates, or circulating tumor cells. If only low cell numbers are collected, then the establishment of the PDO models becomes very difficult.

The third challenge is the calibration of PDO models for clinical decision-making. The ex vivo test results depend on drug concentration or radiation dose and treatment duration. One strategy is to compare drug responses in 2D cell culture or 3D organoids with the response in matched *in vivo* models to determine whether the PDO response is relevant (52). Another method involves comparing PDO results on cancer cells and healthy cells. Both calibration methods are time-consuming and labor-intensive. In addition, drug responses can be quite different due to inter-personal tumor heterogeneity. If we collect data at multiple drug concentrations across a range of tumors to identify the calibrated drug concentration of the PDO models, then we lose the key for personalized medicine.

The timing of testing PDO models can be another problem. The clinical utility of PDO models depends on how quickly results are returned to the clinician. For example, PDO assays that do not require *ex vivo* expansions can typically be completed within several days and still within the treatment course of radiotherapy. However, many solid tumors, especially those starting from core needle biopsies, require expansion for drug or combined RT testing. It often takes several weeks to establish sufficient quantity to perform drug or RT screening prior to decision making (52).

As mentioned previously, organoid models do not include cells of tumor microenvironment (TME). The PDO models are lack of key mediators regulating drug responses. Tumors grown as PDX in nude mice develop a more realistic tumor microenvironment, but interactions with human microenvironmental components, such as immune cells, is still lacking. There is a growing presence of immunotherapy in cancer treatment, incorporating a functional immune microenvironment in the preclinical PDO models is vital (53).

Finally, PDO assays and establishment can be very expensive and labor-intensive. The cost-effective drug or RT testing of PDO or co-culture models in the future may acquire automation and miniaturization procedures.

### Combined Therapy Towards Personalized Oncology

RT combined with chemotherapy, immunotherapy, and targeted therapy can achieve better treatment outcomes for cancer patients. Despite all limitations of the PDO models described in section 5.3, one advantage of functional PDO models is that they may identify active drugs or RT-drug combinations. Even though the combined therapy may suit well for individual patient, in the real world, it faces a significant regulatory challenge. Many RT-drug combinations are never verified in the clinic before, so it would require multi-phase clinical trials to identify the recommended sequences and dosage for radiation and drugs of the combination. Apparently, such an approach is impractical, ineffective, expensive and time-consuming. It is also inconsistent with the goal of identifying novel, individualized combination regimens.

Given the urgent need for combined regimens and regulatory requirements of clinical trials, the PDO platform may be the solution of a timely and effective solution. A two-, three-, or even four-way combination for precision radiotherapy, challenging to verify responses *in vivo*, may be possible and effective for the *ex vivo* PDO models to identify the possible synergistic combination. In the field of clinical RT practice, the combination of cisplatin or immune checkpoint inhibitors can enhance radiosensitivity and treatment efficacy. The PDO platform is suitable for testing novel or optimal combinations of RT with drugs and radiosensitizers (54).

### Future Planning of the PDO Models

In the future there may be a few things to do regarding the PDO models. First, the PDO handling process needs to be standardized and the pre-analytical variability needs to be controlled. Since the handling procedures of viable and unfixed tumor tissue can introduce variability and differences, affecting results of PDO assays downstream. In the future, standard operating procedures (SOP) may be created from the initial acquisition, sample storage, freezing, shipping duration, tumor cell dissociation, culture conditions, to the PDO establishment and maintenance. Making SOP and standardizing the PDO handling process will reduce systematic errors. Also improved communications about the PDO assay between patients, clinicians, and researchers should be established for better patient care.

Second, the comparison of PDO assays need to be rigorous with clinically relevant outcomes. Hopefully with the increasing number of ongoing clinical trials with PDO models, the utility of PDO assays with verified treatment response to various RT-drug combinations may be reported and provided with benefits for personalized medicine. Eventually, with more applications of PDO models in cancer treatment, high-performing assays as standard clinical tools is expected in the future.

Third, PDO models may be adopted more frequently by the pharmaceutical industry for developing new therapeutics or novel combinations. With reliable calibration and quality assurance procedures, PDO models are expected to survey cancer samples and target pathways in a clinical context. Meanwhile, data sharing of the PDO models of different cancer types is important. With enough data collected in the PDO biobank, it’s possible to apply the PDO models’ genomics and response data to predict therapeutic responses for the individual patient as functional assays (55).

## Conclusion

The PDO platform has tremendous potentials for discovering novel drugs or optimizing combined radiation therapies to improve outcomes and reduce toxicities for cancer patients. The surged PDO technology can recapitulate individual patient’ tumor characteristics and facilitate treatment response prediction for personalized radiotherapy. With technology advancement, the PDO models may be incorporated in the mainstream of cancer diagnosis and therapy, and more patients will benefit from the functional PDO assay through precision radiotherapy in the future.

## Author Contributions

YW conceived the idea, searched the literature, wrote the manuscript. YLi and ZS provided insights of radiosensitizers with PDO models, data curation. WD and HY provided advice about the PDO establishment and added a few references. SW and YLiu edited the manuscript and conducted final approval. All authors contributed to the article and approved submitted version.

## Funding

This work is supported by research grants from CAS Key Laboratory of Health Informatics, Shenzhen Institute of Advanced Technology (#2011DP173015), Shenzhen Technology andInnovation Program (JCYJ20210324110210029) and Supported by Research Foundation of Peking University Shenzhen Hospital (JCYJ2020015). 

## Conflict of Interest

The authors declare that the research was conducted in the absence of any commercial or financial relationships that could be construed as a potential conflict of interest.

## Publisher’s Note

All claims expressed in this article are solely those of the authors and do not necessarily represent those of their affiliated organizations, or those of the publisher, the editors and the reviewers. Any product that may be evaluated in this article, or claim that may be made by its manufacturer, is not guaranteed or endorsed by the publisher.
